# Green Synthesis of Thermo-Responsive Hydrogel from Oil Palm Empty Fruit Bunches Cellulose for Sustained Drug Delivery

**DOI:** 10.3390/polym13132153

**Published:** 2021-06-29

**Authors:** Maha Mohammad Al-Rajabi, Yeit Haan Teow

**Affiliations:** 1 Department of Chemical and Process Engineering, Faculty of Engineering and Built Environment, Universiti Kebangsaan Malaysia, Bangi 43600, Selangor Darul Ehsan, Malaysia; p92216@siswa.ukm.edu.my; 2 Research Centre for Sustainable Process Technology (CESPRO), Faculty of Engineering and Built Environment, Universiti Kebangsaan Malaysia, Bangi 43600, Selangor Darul Ehsan, Malaysia

**Keywords:** cellulose hydrogel, thermo-responsive, sustained release, silver sulfadiazine, burn wound

## Abstract

Drug delivery is a difficult task in the field of dermal therapeutics, particularly in the treatment of burns, wounds, and skin diseases. Conventional drug delivery mediums have some limitations, including poor retention on skin/wound, inconvenience in administration, and uncontrolled drug release profile. Hydrogels able to absorb large amount of water and give a spontaneous response to stimuli imposed on them are an attractive solution to overcome the limitations of conventional drug delivery media. The objective of this study is to explore a green synthesis method for the development of thermo-responsive cellulose hydrogel using cellulose extracted from oil palm empty fruit bunches (OPEFB). A cold method was employed to prepare thermo-responsive cellulose hydrogels by incorporating OPEFB-extracted cellulose and Pluronic F127 (PF127) polymer. The performance of the synthesized thermo-responsive cellulose hydrogels were evaluated in terms of their swelling ratio, percentage of degradation, and in-vitro silver sulfadiazine (SSD) drug release. H8 thermo-responsive cellulose hydrogel with 20 w/v% PF127 and 3 w/v% OPEFB extracted cellulose content was the best formulation, given its high storage modulus and complex viscosity (81 kPa and 9.6 kPa.s, respectively), high swelling ratio (4.22 ± 0.70), and low degradation rate (31.3 ± 5.9%), in addition to high t_50%_ value of 24 h in SSD in-vitro drug release to accomplish sustained drug release. The exploration of thermo-responsive cellulose hydrogel from OPEFB would promote cost-effective and sustainable drug delivery system with using abundantly available agricultural biomass.

## 1. Introduction

Drug delivery is a difficult task in the field of dermal therapeutics, particulalry for the treatment of burns, ulcers, and wounds [1]. Conventional drug delivery medium exists in many forms such as, topical liquid (solutions, suspensions, and emulsions), semi-solid formulations (ointments and creams), as well as dry traditional dressings including cotton wool, natural or synthetic bandages and gauzes [2]. However, topical liquid and semi-solid formulations have poor retention on the skin/wound surface where repeated application is required [3]. In addition, the conventional drug delivery media are inconvenient to administer. The dosage forms need to be rubbed into the skin for dispersing the formulations [4], causing pain, inflammation, and irritation [5]. Although dry traditional dressings are easier to use, they do not provide the moist environment that is required for wound healing [2]. On the top of that, conventional methods show uncontrolled drug release profiles, where the drug delivery is rapid and a high topical concentration might result in toxic effects [6]. To address these drawbacks, an alternative drug delivery method—hydrogels—was introduced [2,5].

Hydrogels are basically networks of three-dimensional synthetic or natural polymeric chains, cross-linked by physical or chemical bonds, which are capable of absorbing, swelling, and deswelling (releasing) extensive amount of trapped water, solvent or biological fluids without dissolving into it [7]. The ability of hydrogels to absorb large amounts of water (up to 99% of their weight) [8] and to give a spontaneous response to stimuli imposed on it such as temperature [9], pH [10,11], ionic strength [12], light [13], electric and magnetic fields [14], making them particularly useful for application in various biomedical field, especially as drug delivery media.

Thermo-responsive polymers have the ability to respond to changes in temperature, which make them useful as a dermal drug delivery medium. They exhibit a phase transition at a specific temperature, which causes a sudden change in the solubility. Polymers which become insoluble upon heating are called lower critical solution temperature (LCST) polymers. Whereas polymers which become soluble upon heating are named upper critical solution temperature (UCST) polymers [15]. Pluronic F127 (PF127), a kind of LCST polymer, presents good potential to be used as a hydrogel material as this polymer is in sol-phase below the phase transition temperature (room temperature) and changes to a gel-phase as the temperature is increased above the transition temperature (body temperature) [16]. Due to this sol-gel transition characteristic, it could be poured onto the skin to fill the wound/burn surfaces in sol-phase [17], and transform into a rigid hydrogel. In addition, PF127 was reported to be non-toxic [18] and has been approved by the Food and Drug Administration (FDA) for use as a pharmaceutical ingredient [19]. However, PF127 could not be used alone for hydrogel formation due to its inadequate mechanical strength and mechanical stability [20]. For this, another polymer (or polymers) has to be added to the hydrogel’s network for mechanical strength enhancement. Cellulose is a sustainable, biodegradable, biocompatible, and low toxicity biopolymer. Multiple research works had been developed employing PF127 thermo-responsive polymer with cellulose/cellulose derivatives for hydrogel formation. Kim et al., (2012) synthesized methylcellulose-PF127 hydrogel for anti-cancer docetaxel (DTX) drug delivery. Sustained release pattern of methylcellulose-PF127 hydrogel was significantly enhanced anti-cancer effects of DTX [21]. On the other hand, carboxymethyl cellulose sodium (CMCs)-PF127 hydrogel was developed by Wang et al., (2016) to deliver cortex moutan drug for atopic dermatitis treatment. It was found that the presence of CMCs can appreciably improve the physical properties of PF127 hydrogel, which makes it more suitable for tailored drug loading [19]. However, efforts have been taken to produce more sustainable and green hydrogels, such as using cellulose extracted from agricultural biomass [22], engineered hydrogels based on sustainable cellulose acetate [23], as well as manufacturing of cellulose/alginate monolithic hydrogel for environmental applications [24].

Palm oil industry is considered the vital manufacturing industry in Malaysia [25]. However, this industry has long been associated with negative results such as tropical deforestation, biodiversity loss, and water pollution [26]. In addition, the production of palm oil faces numerous environmental challenges due to its waste generation during the production processes [27]. Oil palm empty fruit bunches (OPEFB) is the most abundant plantation biomass waste. Malaysia produces around 7.78 million tonnes of OPEFB annually [28] and these OPEFB are burned in boilers as a power source. However, this practice had resulted air pollution and raised awareness on the resource management issues [29]. OPEFB consists of 37.3–46.5% cellulose, 25.3–33.8% hemicellulose, and 27.6–32.5% lignin [30]. High cellulose content in OPEFB makes it a good option to be selected as the sustainable polymer to support the network structure of hydrogel for its mechanical strength enhancement.

The objective of this study is to explore a green synthesis method for the development of thermo-responsive cellulose hydrogels using cellulose extracted from OPEFB. The performance of the synthesized thermo-responsive cellulose hydrogels are evaluated in terms of their swelling ratio, percentage of degradation, and in-vitro drug (silver sulfadiazine) release. The exploration of thermo-responsive cellulose hydrogel with the use of cellulose extracted from OPEFB is a good innovation research in utilizing agricultural waste as a new source of cellulose in drug delivery medium. Long-term economic, social, and environmental sustainability could be ensured with the use of cost-effective and abundantly available raw materials.

## 2. Materials and Methods

### 2.1. Materials

Oil palm empty fruit bunches (OPEFB) was collected from the Tennamaram palm oil mill located at Bestari Jaya ( Selangor Darul Ehsan, Malaysia). NaOH and hydrogen peroxide (30 w/w%) were obtained from Classic Chemicals Sdn. Bhd. (Selangor Darul Ehsan, Malaysia). Formic acid (98–100 w/w%) was purchased from Thermo Fisher Scientific (Waltham, MA, USA). Ethanol (99.5 v/v%) was supplied by Scienfield Expertise PLT (Selangor Darul Ehsan, Malaysia). Pluronic F127 (PF127) (molecular weight: 12,600 Da, 70 w/w% polyethylene oxide (PEO)) was obtained from Sigma-Aldrich (Hamburg, Germany). Silver sulfadiazine (SSD) (>98 w/w%) was supplied by Tokyo Chemical Industry (Tokyo, Japan). All chemicals were ACS grade and used as purchased.

### 2.2. Synthesis of Thermo-Responsive Cellulose Hydrogel

The cellulose extraction process was adapted and modified from the study conducted by Nazir et al. (2013) [31]. Firstly, OPEFB was washed with 1 w/v% detergent until the rinse water turned colorless. Next, washed OPEFB was dried at 100 ± 2 °C until a constant weight was obtained. Following, dried OPEFB was cut, and sieved using 1.18 mm opening mesh sieve. Consequently, dry OPEFB was de-waxed using 70 v/v% ethanol in soxhlet extraction apparatus for 6 h at 78 ± 2 °C. The OPEFB fibers were then washed with deionized (DI) water and dried at 100 ± 2 °C. 3 w/v% NaOH solution was added to de-waxed OPEFB fibers and heated to 121 °C for 1 h for delignification. Acid treatment was then started by soaking 10 g of delignified OPEFB fibers in 200 mL of 20 v/v% formic acid and 10 v/v% hydrogen peroxide mixture. The mixture was heated to 85 °C for 2 h. Finally, the light yellow OPEFB biocellulose fibers were bleached by suspending in 10 v/v% hydrogen peroxide with pH 9 at 60 °C for 90 min. Next, OPEFB biocellulose fibers were rinsed with DI water until neutral pH was obtained and dried in an oven at 60 ± 2 °C. Cold method was employed for the synthesis of thermo-responsive cellulose hydrogel [32]. Typically, 15–35 w/v% of PF127 was dissolved in DI water and kept in refrigerator (2–8 °C) for 20 h until complete dissolution was achieved. Next, 0.0–3.0 w/v% of extracted cellulose fibers were added into PF127 aqueous solution. The mixed solution was continuously stirred at 200 rpm for 7 days at low temperature (2–8 °C) to obtain a homogeneous thermo-responsive cellulose hydrogel. The formula of thermo-responsive cellulose hydrogel developed in this study was summarized in Table 1.

### 2.3. Characterization of Thermo-Responsive Cellulose Hydrogel

#### 2.3.1. Sol-Gel Transition Temperature (SGTT)

Tube inversion method was applied to determine the sol-gel transition temperature of the synthesized thermo-responsive cellulose hydrogel. 1 mL of thermo-responsive cellulose hydrogel was added into a glass vial and heated from 15 °C to 90 °C at heating rate of 1 °C/min for 15–25 °C and at heating rate of 5 °C/min for 25–90 °C in a water bath [33]. The flow behaviour of the thermo-responsive cellulose hydrogel at 4 °C, 20 °C, and 37 °C was observed by tilting the vial at different temperature: 4 °C (storing temperature in fridge), 20 °C (ambient temperature), and 37 °C (human body temperature) [34]. The flow behaviour of the thermo-responsive cellulose hydrogel was described in four categories: liquid free flow, slow flow (high viscosity), hard to flow (weak gel), solid-like behavior, and non-free flow. The temperature at which the thermo-responsive cellulose hydrogel was immobile was recorded as the gelation transition temperature [19]. Triplicate SGTT test was conducted for each thermo-responsive cellulose hydrogel and the average SGTT value was reported with standard deviation. Thermo-responsive cellulose hydrogels with selective flow behaviour were chosen as the medium for drug delivery. It was further characterized on its functional group, surface morphology and structure, and rheological property.

#### 2.3.2. Functional Group

The functional groups presence on the synthesized thermo-responsive cellulose hydrogel were ascertained using fourier transform infrared spectroscopy (FTIR), Nicolet 6700 (Thermo Scientific, Waltham, MA, USA) at attenuated total reflectance (ATR) mode for wavenumber ranging from 500 to 4000 cm^−1^ and under 32 scans.

#### 2.3.3. Surface Morphology and Structure

Top surface morphology and cross-section structure of the synthesized thermo-responsive cellulose hydrogel were observed using field emission scanning electron microscope (FESEM), Merlin Compact (Carl Zeiss, Jena, Germany). Firstly, the synthesized thermo-responsive cellulose hydrogel was soaking in liquid nitrogen, fractured, and mounted vertically onto the sample holder. It was then coated with a thin layer of iridium using the vacuum sputter coater, Q150T S (Quorum Technologies, Lewes, East Sussex, UK). The cross-section structure of thermo-responsive cellulose hydrogel was observed under 100× magnification. Whereas, the top surface morphology of thermo-responsive cellulose hydrogel was observed under 2.50k× magnification.

#### 2.3.4. Rheological Property

Rheological property of the synthesized thermo-responsive cellulose hydrogel was measured using Physica MCR301 Rheometer (Anton Paar, Graz, Austria). The heating rate of rheometer was set constant at 1 °C/min with temperature range between 15 °C (ambient temperature under non-physiological condition) and 37 °C (human body temperature under physioligical condition). Storage modulus (G′) and complex viscosity (η*) values were recorded as the function of temperature at fixed angular frequency of 10 1/s. G′ is a measure of energy stored and recovered per deformation cycle. High G′ value reflects elastic or highly structured thermo-responsive cellulose hydrogel [35]. On the other hand, η* is an overall resistance to deformation, and it is a function of complex shear modulus (G*) [36]. G* is sum of the elastic and viscous components of the thermo-responsive cellulose hydrogel, represented by G′ and loss modulus (G″), respectively. η* and G* are described in Equations (1) and (2), respectively.

(1) $$\eta^{*}=\frac{G^{*}}{\omega }$$

where ω is the angular frequency (rad/s):

(2) $$G^{*}=\;G^{\prime }+{\mathrm{iG}}^{\prime \prime }$$

where i is the complex number (√-1).

### 2.4. Performance of Thermo-Responsive Cellulose Hydrogel

#### 2.4.1. Swelling and Degradation

Dried thermo-responsive cellulose hydrogel (0.2 g) was weighed and allowed to swell in 2 mL DI water at room temperature [37]. The thermo-responsive cellulose hydrogel after 15 min, 30 min, 60 min, 120 min, 180 min, and 240 min of swelling was superficially dried with filter paper and weighed. DI water was added after each sampling to maintain the DI water volume throughout the swelling and degradation test. Thermo-responsive cellulose hydrogel swelling ratio was calculated using Equation (3), whereas the percentage of thermo-responsive cellulose hydrogel degradation was calculated using Equation (4) [37]:

(3) $$\mathrm{Swelling}\;\mathrm{ratio}\;=\left[\frac{W_{t}-{\;W}_{0}}{W_{0}}\right]$$

where W_t_ is the weight of thermo-responsive cellulose hydrogel after swelling at time t (g) and W_0_ is the initial weight of dry thermo-responsive cellulose hydrogel (g):

(4) $$\mathrm{Percentage}\;\mathrm{of}\;\mathrm{degradation}\;\left(\%\right)=\left[\frac{{\mathrm{Ws}}_{0}-W_{d}\;}{{\mathrm{Ws}}_{0}}\right]\times 100\;\%\;$$

where W_d_ is the weight of thermo-responsive cellulose hydrogel after saturation (maximum swelling) at time t (g) and Ws_0_ is the maximum weight of thermo-responsive cellulose hydrogel in the swelling test (g).

#### 2.4.2. In-Vitro Drug Delivery Study

SSD (100 mg) was loaded into thermo-responsive cellulose hydrogel (10 g) in sol-phase to simulate the commercial SSD formulation [38]. The thermo-responsive cellulose hydrogel loaded with SSD was then stirred at 200 rpm for 1 h under low temperature (2–8 °C) to obtain a homogeneous drug-loaded thermo-responsive cellulose hydrogel [34].

Vertical diffusion cell, Copley HDT 1000 (Copley, County Durham, UK) was used for SSD release from the thermo-responsive cellulose hydrogel. 0.25 v/v% ammonia in phosphate buffer solution at pH 7.4 was the receptor medium [39,40]. On the other hand, 0.45 µm regenerated cellulose filter pre-soaked in receptor medium for 30 min was used as membrane. Firstly, vertical diffusion cell was filled with 11 mL of receptor medium. It was then heated to 32 °C under continuous stirring at 600 rpm [41]. Thereafter, 0.2 g of drug-loaded thermo-responsive cellulose hydrogel was placed at the drug donor chamber and heated in an oven at 32 °C for 10 min to ensure complete solidification. Following, the pre-soaked regenerated cellulose filter was then placed between the drug-loaded thermo-responsive cellulose hydrogel and heated receptor medium in vertical diffusion cell to maintain a close contact between thermo-responsive cellulose hydrogel and receptor medium, allowing SSD releases from thermo-responsive cellulose hydrogel to receptor medium [42].

Receptor medium (1 mL) was withdrawn from the vertical diffusion cell at 15 min intervals for the first hour, followed by 1 h intervals for the following 25 h. Once the receptor medium was withdrawn, an equal volume of fresh receptor medium was added into the vertical diffusion cell. SSD concentration in sampled receptor medium was analyzed by UV-visible spectrophotometer, Genesys 10S UV-VIS (Thermo Scientific, Waltham, MA, USA) at the wavelength of 260 nm. Cumulative percentage of SSD release from thermo-responsive cellulose hydrogel was calculated using Equation (5) [43]:

(5) $$\mathrm{Cumulative}\;\mathrm{percentage}\;\mathrm{of}\;\mathrm{SSD}\;\mathrm{release}\;\left(\%\right)=\left[\frac{C_{t}\;V+v\sum_{1}^{t-1}C_{t}\;}{W_{D}}\right]\times 100\;\%\;$$

where C_t_ is the concentration of SSD released at time t (mg/mL), V is the volume of receptor medium (mL), v is volume of receptor medium being withdrawn (mL), and W_D_ is the initial amount of SSD in thermo-responsive cellulose hydrogel (mg).

#### 2.4.3. Kinetic Study of Drug Release 

Several kinetic models were applied to describe the kinetic governing SSD release from thermo-responsive cellulose hydrogel.

(i) Zero-Order Model

Zero-order model assumes the area of dosage form (thermo-responsive cellulose hydrogel) does not change significantly with time. This model supports slow release of drug as the dug loaded into dosage form is not disaggregate. The zero-order model is depicted by Equation (6) [44]:

(6) $$Q_{t}=Q_{0}+k_{0}t\;$$

where Q_t_ is the percentage of drug released at time t (%), Q_0_ is the initial percentage of drug in receptor medium (%), k_0_ corresponds to Zero-order model constant (1/h), and t is the sampling time of receptor medium (h).

(ii) First-Order Model

First-order model often use to describe the absorption and release of water soluble drug. The rate of drug release is depends on its initial concentration. First-order model is depicted by Equation (7) [45]:

(7) $${\mathrm{LogQ}}_{t}={\mathrm{LogQ}}_{0}-\frac{k_{1}}{r}t\;$$

where k_1_ corresponds to First-order model constant (1/h) and r is the conversion factor (2.303).

(iii) Higuchi Model

The Higuchi model describes the release of soluble and sparingly soluble drug. Higuchi model assumes that: (i) the initial drug concentration in dosage form is higher than drug solubility, (ii) drug only spread in one dimension, (iii) drug diffusivity does not change, (iv) sink condition is achieved where the receptor medium has high capacity to dissolve the drug [41], Higuchi model is depicted by Equation (8) [46]:

(8) $$Q_{t}=k_{H}t^{1/2}\;$$

where k_H_ is Higuchi model constant (1/h^1/2^).

#### 2.4.4. Mechanism of Drug Release

Korsmeyer-Peppas model is a generalized model of Higuchi model to describe drug release mechanism from polymeric dosage form where erosion and/or dissolution of the dosage form occurs. Korsmeyer-Peppas model is depicted by Equation (9) [47]:

(9) $$Q_{t}=k_{r}t^{n}$$

where k_r_ is the Korsmeyer-Peppas model constant (1/(h)^n^), n is the diffusion exponent indicates the mechanism of drug molecules transport from the dosage form.

### 2.5. Statistical Analysis

Each experiment was independently repeated in triplicate, and the results were presented as mean ± standard deviation (SD). SD was calculated using the STDEV formula in Excel.

## 3. Results and Discussion

### 3.1. Characterization of Thermo-Responsive Cellulose Hydrogel

#### 3.1.1. Sol-Gel Transition Temperature (SGTT)

**Figure 1:**
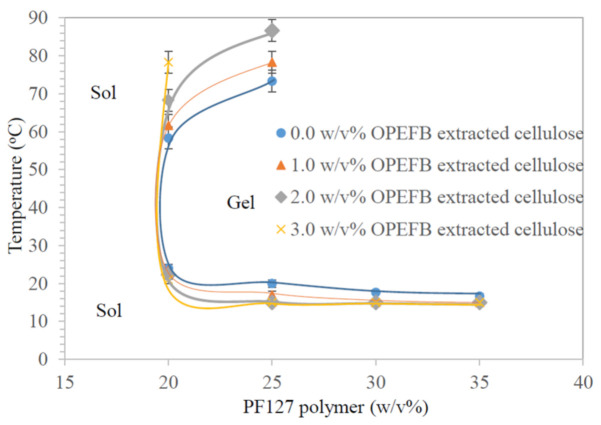
Phase diagram of the thermo-responsive cellulose hydrogels.

The SGTT and flow behavior of the thermo-responsive cellulose hydrogels are summarized in Table 2, while Figure 1 shows the phase diagram of the synthesized thermo-responsive cellulose hydrogels. Generally, SGTT consists of a lower critical solution temperature (LCST) and upper critical solution temperature (UCST). LCST is the temperature at which the thermo-responsive cellulose hydrogel in sol form transforms into gel form due to an increase of temperature. On the other hand, UCST is the temperature at which the thermo-responsive cellulose hydrogel in gel form transform back into gel form by further increase in temperature [48].

As presented in Table 2, SGTT is not measurable for H1-H4 thermo-responsive cellulose hydrogels. PF127 consists of hydrophilic PEO and hydrophobic polypropylene oxide (PPO) blocks (unimers) arranged in (PEOx-PPOy-PEOx) tri-block structure [49]. Unimers are water soluble, hence it prevents micelle formation, packing and entanglement for solid-like hydrogel formation at low PF127 percentage (15 w/v%) [50]. Whereas, LCST of H5-H20 thermo-responsive cellulose hydrogels is decreased with the increasing of PF127 percentage at constant OPEFB extracted cellulose percentage; while UCST of H5-H20 thermo-responsive cellulose hydrogels shows an opposite trend. This could be explained with the aid of Figure S1. When the PF127 percentage is exceeding critical micelle concentration (CMC) and/or critical micelle temperature (CMT), unimers are aggregate to form spherical micelles with hydrophobic PPO blocks as central core surrounded by hydrophilic PEO chains. This process is called micellization. The PF127 micelles are stable in water with PEO hydrophilic chains in-contact with water molecules through the formation of hydrogen bonds [17,51]. More PF127 micelles in the thermo-responsive cellulose hydrogel at higher PF127 percentage will drive the aggregation of unimers and promote for the formation solid-like hydrogel at lower temperature [52]. This explains the decrease of LCST with the increase of PF127 percentage at constant OPEFB extracted cellulose percentage.

When the temperature is raised above the LCST, hydrogen bonds between water molecules and PEO hydrophilic chains of PF127 are broken due to dehydration of hydrophilic PEO chains [53]. The occurrence of segregation between PEO chains and PPO blocks is attributed by shrinking of the gel phase structure. As hydrophobic PPO associations are dominany, this leads to solid-like hydrogel formation [52]. An increasing PF127 percentage in a thermo-responsive cellulose hydrogel formulation results in greater aggregation in the form of solid-like hydrogel. As such, a higher UCST is required to loosen the PF127 micelle interaction for transforming it from a gel state to a sol state [54]. A similar trend was observed for thermo-responsive cellulose hydrogels synthesized with different OPEFB -extracted cellulose percentages at constant PF127 percentage. The LCST of thermo-responsive cellulose hydrogel is decreased while the UCST of thermo-responsive cellulose hydrogel is increased with the increasing of OPEFB extracted cellulose percentage in formulation. This is because OPEFB-extracted cellulose could bind with hydrophilic PEO chains through intermolecular hydrogen bonding [55]. This would promote dehydration of hydrophilic PEO chains as the bonding between OPEFB-extracted cellulose and hydrophilic PEO chains decreases the hydrogen bonding between hydrophilic PEO chains and water molecules. This will cause an increase in entanglement of adjacent P127 micelles, thus promoting the occurrence of gelation at a lower temperature [55]. However, the UCST of H12-H20 thermo-responsive cellulose hydrogels is not detected. This is estimated to happen at temperatures higher than 90 °C.

For the aspect of flow behaviour, pristine hydrogel (H1) does not show changes in flow behavior at different temperatures but thermo-responsive cellulose hydrogels did. Generally, the flow behaviour of thermo-responsive cellulose hydrogels was changed from liquid free flowing to slow flow, hard flowing, and finally non-free flow with the increase of both OPEFB-extracted cellulose percentage and/or PF127 percentage. The change of flow behavior is attributed by higher viscosity in thermo-responsive cellulose hydrogel formulations [56]. The ideal thermo-responsive cellulose hydrogel should be free flowing under the preparation conditions (4 °C) to ease the drug loading process [57], easy flow at room temperature (20 °C) for applying onto the skin [17] and form a non-flowing solid hydrogel at body temperature (37 °C) for its application as a drug delivery medium [17]. As presented in Table 2, H5-H8 thermo-responsive cellulose hydrogels with 20 w/v% PF127 fulfilled the prescribed properties and thus are suitable for use as a drug delivery medium. With this, H5-H8 thermo-responsive cellulose hydrogels were further characterized regarding their functional groups, surface morphology and structure, and rheological properties.

#### 3.1.2. Functional Groups

**Figure 2:**
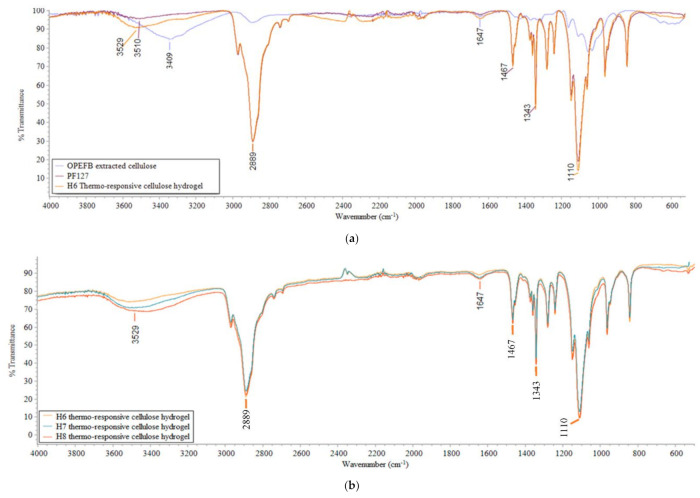
FTIR spectrum of (**a**) OPEFB extracted cellulose, PF127, H6 thermo-responsive cellulose hydrogel (**b**) H6-H8 thermo-responsive cellulose hydrogels.

The FTIR spectrum of OPEFB-extracted cellulose, PF127, and H6-H8 thermo-responsive cellulose hydrogels are shown in Figure 2. As illustrated in Figure 2a, a broad peak was observed for H6 thermo-responsive cellulose hydrogel at 3529 cm^−1^, confirming the presence of O–H stretching vibrations [58]. On the other hand, the peak at 1647 cm^−1^ was attributed by O–H bending [59]. The intensity of the O–H stretching vibration peak and O–H bending peak for H6 thermo-responsive cellulose hydrogel was the highest compared to PF127 and OPEFB-extracted cellulose. This indicates that H6 thermo-responsive cellulose hydrogel has higher hydrophilicity than its base material (PF127) due to the alteration of its properties by hydrophilic OPEFB-extracted cellulose. The influence of hydrophilic OPEFB-extracted cellulose could also reflected by thermo-responsive cellulose hydrogels at different OPEFB-extracted cellulose percentages. By varying the OPEFB extracted cellulose percentage from 1 to 3 w/v% for H6-H8 thermo-responsive cellulose hydrogels, the intensity of the O–H stretching vibration peak and O–H bending peak was increased.

On the other hand, there was a slight shift of the O–H stretching vibration peak in thermo-responsive cellulose hydrogels compared to both PF127 and OPEFB-extracted cellulose. The shift of the O–H stretching vibration peak to a higher wavenumber was probably due to the increase of O–H bond strength [60]. Strong O–H bond strength in thermo-responsive cellulose hydrogels indicates strong intermolecular hydrogen bonding between PF127 and OPEFB-extracted cellulose.

#### 3.1.3. Surface Morphology and Cross-Section View

**Figure 3:**
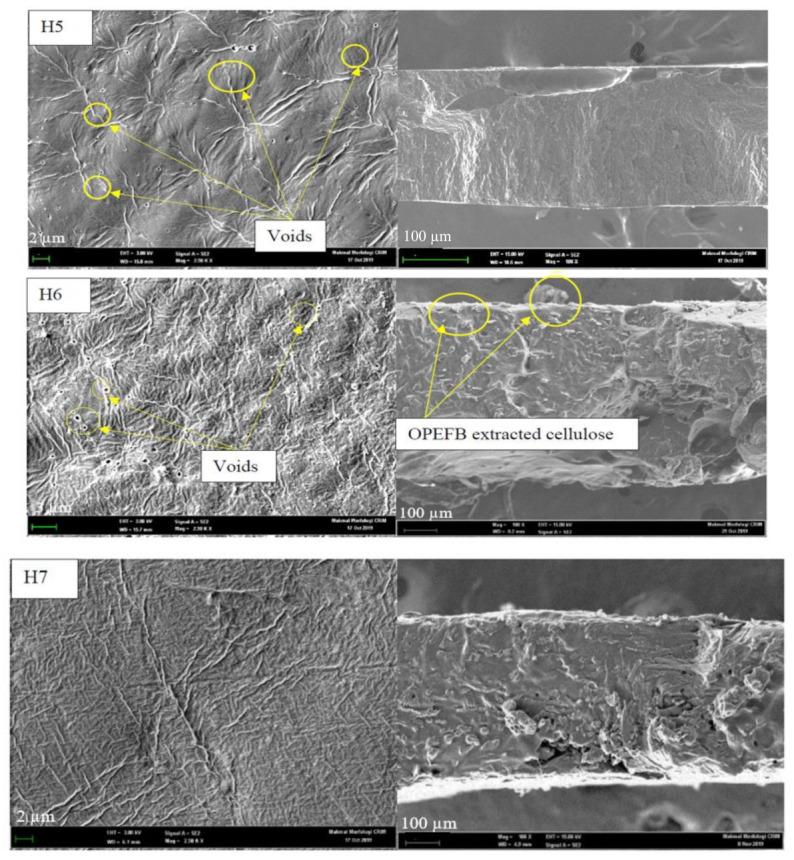

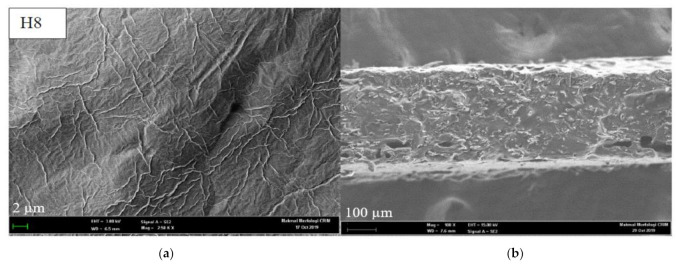
(**a**) Surface morphology and (**b**) cross-sectional view of thermo-responsive cellulose hydrogels at the magnification of 2.50k× and 100×, respectively.

Figure 3 presents the surface morphology and cross-sectional view of H5-H8 thermo-responsive cellulose hydrogels. Generally, the surface of thermo-responsive cellulose hydrogels was not smooth, with fibers deposited on the thermo-responsive cellulose hydrogels’ surface. H5 and H6 thermo-responsive cellulose hydrogels were associated with some voids at a diameter range between 0.22–0.76 µm and 0.22–0.66 µm, respectively. In comparison, the surfaces of H7 and H8 thermo-responsive cellulose hydrogels were smoother with no voids on the surface. The smoother H7 and H8 thermo-responsive cellulose hydrogels’ surface was possibly due to a stronger cross-linking effect between OPEFB-extracted cellulose and PF127 at higher OPEFB-extracted cellulose percent. High cross-linking density was proven to be able to produce a dense and smooth surface [61]. A smoother hydrogel surface was observed by Rasoulzadeh and Namazi (2017) with the increase of graphene oxide content in carboxymethyl cellulose hydrogel and attributed to stronger hydrogen bonding interactions between graphene oxide and carboxymethyl cellulose [62].

On the other hand, the cross-section morphology of H6-H8 thermo-responsive cellulose hydrogels were rougher than that of pristine thermo-responsive hydrogel, H5. Similar to surface morphology, it was probably due to the presence of OPEFB extracted cellulose in thermo-responsive hydrogel’s matrix. However, there was not significant aggregation of OPEFB-extracted cellulose fibers in thermo-responsive hydrogel matrix. Hydrophilic-hydrophilic interaction between OPEFB-extracted cellulose and PF127 had resulted well distribution of OPEFB-extracted cellulose fiber in the thermo-responsive cellulose hydrogels’ matrix.

#### 3.1.4. Rheological Property

**Figure 4:**
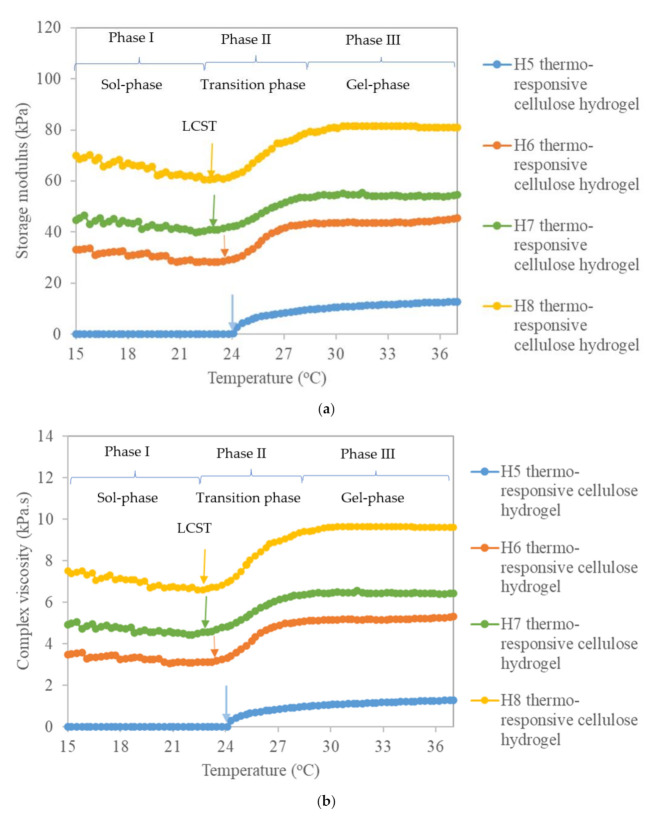
(**a**) Storage modulus (G′) and (**b**) complex viscosity (η*) of thermo-responsive cellulose hydrogels as a function of temperature.

Figure 4 presents the rheological behavior (storage modulus (G′) and complex viscosity (η*)) of thermo-responsive cellulose hydrogels as a function of temperature. As shown in Figure 4, both the G′ and η* values of thermo-responsive cellulose hydrogels are temperature-dependent, confirming the thermo-responsive behaviour of the hydrogels. At phase I where the temperature is between 15 °C and LCST, the pristine thermo-responsive hydrogel H5 recorded a zero value for both G′ and η*. Whereas, the G′ and η* values were increased with the increase of OPEFB-extracted cellulose percent in thermo-responsive hydrogel formulations. This indicates an increase of the elasticity and viscosity of thermo-responsive cellulose hydrogels with the increase of OPEFB-extracted cellulose percent in the thermo-responsive hydrogels’ formulation. The results agree well with the flow behavior of thermo-responsive cellulose hydrogels, where it changed from liquid free flow to slow flow, hard flowing, and finally non-free flow with the increase of OPEFB- extracted cellulose percent due to higher elasticity and viscosity of the formulation. A viscous thermo-responsive cellulose hydrogel at room temperature is important for a drug delivery medium in topical applications. A viscous thermo-responsive cellulose hydrogel is needed for it to stay on the skin and not flow off after application [63]. On the other hand, both G′ and η* values of thermo-responsive cellulose hydrogels were decreased with the increase of temperature in phase I. As the temperature increased, the thermo-responsive cellulose hydrogel structure was deformed and transformed into a sol-phase. The decrease of viscosity therefore led to the reduction of both G′ and η* values.

In phase II, where the temperature is between LCST and 30 °C, G′ and η* values of thermo-responsive cellulose hydrogels were increased with the increase of temperature, revealing the transition of thermo-responsive cellulose hydrogels from sol-phase to gel-phase. As the temperature increased in this transition phase (phase II), PF127 micellization and its interaction with OPEFB-extracted cellulose occurred, leading to the formation of dense, solid-like network with predominant elastic properties [52,64]. It is therefore gives larger G′ and η* values at higher temperature. However, in phase III where the temperature is between 30 °C and 37 °C, G′ and η* values reached a plateau. This signified that the thermo-responsive cellulose hydrogels’ network does not undergo further structural transition at temperatures above 30 °C. The stability of thermo-responsive cellulose hydrogel with gel-like behaviour [65] during phase III is desired for thermo-responsive cellulose hydrogel application on human skin. On top of that, the plateau values of G′ and η* at phase III were used to indicate the strength of thermo-responsive cellulose hydrogels and their cross-linking density [66]. The G′ and η* values of thermo-responsive cellulose hydrogels ranged between 48–81 kPa and 5.6–9.6 kPa.s, respectively. Thus was higher than that of a thermo-responsive nanocrystal cellulose hydrogel (G′ value of 15–40 kPa) [56] and a thermo-responsive pentablock (polyethylene glycol-polycaprolactone polylactide-polycaprolactone-polyethylene glycol) one (η* value of 1.1–2.9 kPa.s) [67], validating the favorable use of thermo-responsive cellulose hydrogel as a drug delivery medium.

### 3.2. Performance of Thermo-Responsive Cellulose Hydrogel 

#### 3.2.1. Swelling and Degradation

**Figure 5:**
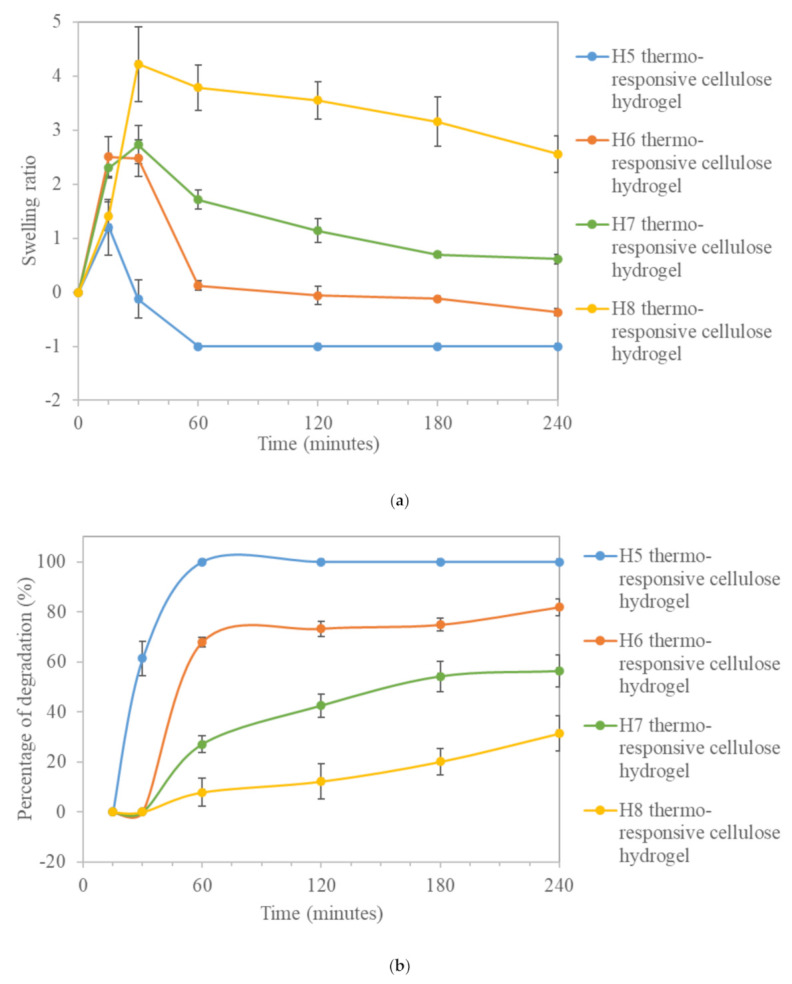
(**a**) Swelling ratio and (**b**) percentage degradation of thermo-responsive cellulose hydrogels.

Figure 5 shows the swelling ratio and the percentage degradation of thermo-responsive cellulose hydrogels as a function of time after immersing them in DI water at room temperature. As presented in Figure 5a, the swelling ratio of thermo-responsive cellulose hydrogel was increased with time and reaches a maximum at 15 min for pristine thermo-responsive hydrogel, H5 and at 30 min for H6-H8 thermo-responsive cellulose hydrogels. At the beginning of swelling process, hydration of the thermo-responsive cellulose hydrogels occurred. Water molecules were bonded to hydrophilic O–H groups in the thermo-responsive cellulose hydrogels, either by adsorption into the pores and/or into the thermo-responsive cellulose hydrogel’s matrix [68]. Besides, the swelling ratio was increased with the increase of OPEFB-extracted cellulose percentage in the thermo-responsive hydrogels’ formulation. Thermo-responsive cellulose hydrogels with higher weight percent of OPEFB-extracted cellulose exhibit a stronger affinity towards water absorption and are able to retain a higher fraction of water within their structure. This is mainly due to the presence of more O–H groups in hydrogel matrix as depicted by the higher O–H functional group intensity seen in the corresponding FTIR spectrum (Figure 2). A high swelling ratio is desirable for a thermo-responsive cellulose hydrogel to achieve a high drug loading capacity [69] and to maintain the moisture content on targeted skin area to provide cooling and soothing effects and reduce pain [70].

The swelling mechanism of thermo-responsive cellulose hydrogel could be explained by the Flory–Rehner theory [71], which posits that a cross-linked polymer swells in a solvent due to the swelling pressure, π. The swelling pressure is the summation of osmotic pressure (π_osmotic_) and an elastic pressure (π_elastic_), as described in Equation (10):

(10) $$\;\pi =\pi_{\mathrm{osmotic}}+{\;\pi }_{\mathrm{elastic}}$$

As shown in Figure S2, when a dry thermo-responsive cellulose hydrogel was immersed in DI water, the thermo-responsive cellulose hydrogel started to swell by drawing in DI water due to the π_osmotic_ difference between thermo-responsive cellulose hydrogel and DI water [72]. At first, π_osmotic_ dominates the swelling pressure [72,73]. As swelling proceeds, incoming DI water molecules in the thermo-responsive cellulose hydrogel will exert pressure on the thermo-responsive cellulose hydrogel’s chains. The pressure exerted on the thermo-responsive cellulose hydrogel’s chains stretches the hydrogel’s chains by pushing the cross-links apart [74]. Conversely, the thermo-responsive cellulose hydrogel’s chains would resist the deformation, imposing a π_elastic_ in opposite direction of π_osmotic_ [74]. The swelling process was continued until π_elastic_ and π_osmotic_ were in equilibrium [72]. The swelling ratio reaches a maximum at this stage where the thermo-responsive cellulose hydrogel’s chains will not further swell [75]. After reaching the maximum value, the swelling ratio starts to decrease and reaches saturation. A further increase of water content in thermo-responsive cellulose hydrogel would result in degradation and dissolution of the thermo-responsive cellulose hydrogel [68,76]. The swelling ratio values of the thermo-responsive cellulose hydrogels ranged between 1.20–4.22. This was higher than that of carbon nano-onions-reinforced natural protein nanocomposite hydrogels (swelling ratio value of 0.42–0.65) [10], validating the favorable use of thermo-responsive cellulose hydrogels as drug delivery media.

As shown in Figure 5b, percentage degradation of thermo-responsive cellulose hydrogels increased with time and eventually reached a plateau. The pristine thermo-responsive hydrogel H5 was degraded completely within 1 h. On the other hand, thermo-responsive cellulose hydrogels H6-H8 were only partially degraded after 4 h of degradation study. The degradation ability of the thermo-responsive cellulose hydrogels was decreased with the increase of OPEFB-extracted cellulose percentage in the thermo-responsive hydrogels’ formulation. The higher cross-linking density of thermo-responsive hydrogel’s attributed to a higher OPEFB-extracted cellulose percentage in its formulation produces strong a thermo-responsive cellulose hydrogel structure as proven by the rheological properties discussed in Section 3.1.4. The strong thermo-responsive cellulose hydrogel structure therefore prolonged the degradation process. Specifically, drug release from a drug delivery medium is controlled by the rate of degradation of the drug delivery medium. The duration of drug release can be regulated by tailoring the thermo-responsive cellulose hydrogel’s degradation rate [77]. With this, H8 thermo-responsive cellulose hydrogel with high swelling ratio and low degradation rate is considered the most suitable drug delivery medium to retain high drug loading capacity with sustained drug release.

#### 3.2.2. In-Vitro Drug Delivery Study

**Figure 6:**
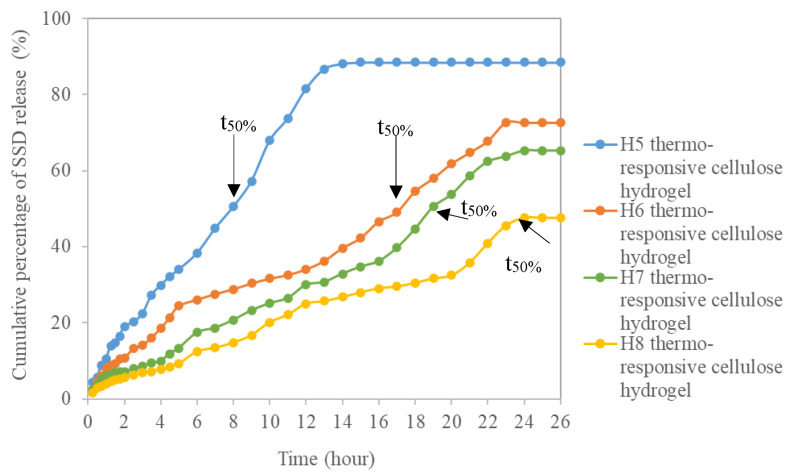
Cumulative percentage of SSD release from thermo-responsive cellulose hydrogels.

The cumulative percentage of SSD released from the thermo-responsive cellulose hydrogels as a function of time is depicted in Figure 6. The half-life time of SSD release (t_50%_) is the time at which the mass fraction of SSD released reached 50%. t_50%_ for the pristine thermo-responsive hydrogel H5 was recorded at 8 h. It was increased to 17 h, 19 h, and 24 h for H6, H7, and H8 thermo-responsive cellulose hydrogels, respectively. Higher weight percent of OPEFB-extracted cellulose in the thermo-responsive cellulose hydrogels’ formulation leads to strong interlocking of SSD within the thermo-responsive cellulose hydrogel network as a result of the greater interaction between PF127 and OPEFB-extracted cellulose, therefore, prolonging the sustained released of SSD. The t_50%_ values of thermo-responsive cellulose hydrogels range between 8 to 24 h were comparatively higher than commercial SSD cream and cubosomes aloe vera SSD hydrogel with t_50%_ value of 5 h and 8 h, respectively [78]. This is an impressive result proving that thermo-responsive cellulose hydrogel is a promising drug delivery medium which is able to accomplish sustained drug release reducing the fluctuation of drug levels during administration. Extended-release drug delivery media are an attractive therapeutic option for the treatment of complex chronic diseases, such as cancer [79].

On top of that, the concentration of the released drug at the targeted tissue should attain the minimum inhibitory concentration (MIC) [68] where it is the minimum concentration of antimicrobial drug needed in plasma to inhibit the growth of micro-organisms [80]. Table S1 summarizes the concentration of SSD released from thermo-responsive cellulose hydrogels at different time intervals. As shown in Table S1, the concentration of SSD released from the thermo-responsive cellulose hydrogels was above 18 mg/L, the MIC for *Pseudomonas aeruginosa* [81] after 1, 2, 4.5, and 6 h of in-vitro release for H5, H6, H7, and H8 thermo-responsive cellulose hydrogels, respectively. Therefore, thermo-responsive cellulose hydrogel delivering SSD could be effective against *Pseudomonas aeruginosa*, one of the most common causes of burn wound infections [82].

#### 3.2.3. Kinetic and Mechanism Study of Drug Release

Figure S3a–d show linear regressions of the kinetic models applied for our drug release study. The models’ constants and correlation coefficient, R^2^ are summarized in Table 3. A high R^2^ value obtained from a specific kinetic model indicates the most appropriate kinetic model in explaining the release of the drug [82]. Among all kinetic models, the zero-order model has the highest R^2^ value for all thermo-responsive cellulose hydrogel formulations in the drug release study. This indicates the zero-order model was the best fit in describing the SSD release mechanism from the thermo-responsive cellulose hydrogels. Based on the zero-order model, the release rate of thermo-responsive cellulose hydrogels is constant over time and independent of the drug concentration [83], in contrast to the other kinetic models, where the release rate is drug concentration dependent, and in which the higher the drug concentration, the faster the release rate [82]. The zero-order model constant, k_0_, is used to reflect the release rate. As presented in Table 3, the k_0_ value decreased with increasing OPEFB-extracted cellulose percent in the thermo-responsive hydrogel’s formulation. H8 thermo-responsive cellulose hydrogel with the highest weight percent of OPEFB-extracted cellulose exhibited the lowest k_0_, meaning it had a longer SSD release than other formulations. This agrees well with in-vitro drug delivery study outcomes described in Section 3.2.2.

On the other hand, the linear regression of the Korsmeyer-Peppas model applied for drug release mechanism study is presented in Figure S3d. The diffusion exponents (n) of the Korsmeyer-Peppas model obtained from the plots are summarized in Table 3. The n values of the H5-H8 thermo-responsive cellulose hydrogels range between 0.671 and 0.746. An n value ranging between 0.5 and 1 indicates that the SSD release mechanism of thermo-responsive cellulose hydrogels followed a non-Fickian diffusion [84]. As explained by non-Fickian diffusion, the SSD release rate is controlled by both SSD drug diffusion and thermo-responsive cellulose hydrogel erosion [83]. The SSD drug release mechanism could be explained with the aid of Figure S4. Initially, SSD-loaded thermo-responsive cellulose hydrogel applied onto a wound surface is in close contact with wound exudates. Then, SSD molecules are transferred from the thermo-responsive cellulose hydrogel to the wound surface through diffusion due to this close contact. The driving force for SSD release by diffusion is the SSD concentration gradient between the thermo-responsive cellulose hydrogel and the wound surface [85]. Besides SSD diffusion, SSD release from thermo-responsive cellulose hydrogels is also contributed by physical dissolution of the thermo-responsive cellulose hydrogel as a result of its degradation [86]. As a thermo-responsive cellulose hydrogel’s matrix is eroded, entrapped SSD molecules are set free and released to the wound surface. The degradation of thermo-responsive cellulose hydrogels contributing to SSD release rate had been validated in the degradation study presented in Section 3.2.1.

## 4. Conclusions

In conclusion, this study had successfully explored a green synthesis method for the development of thermo-responsive cellulose hydrogels from OPEFB, which is the most produced agricultural biomass in the palm oil industry. This study can not only solve the problem of OPEFB biomass waste, but also produce thermo-responsive cellulose hydrogels with superior performance in comparison with literature data and commercial drug delivery media. Among the different thermo-responsive cellulose hydrogel formulations, the H8 thermo-responsive cellulose hydrogel with 3 w/v% OPEFB-extracted cellulose is the best thermo-responsive cellulose hydrogel formulation in this study as it exhibited a high storage modulus and complex viscosity (81 kPa and 9.6 kPa.s, respectively), high swelling ratio (4.22 ± 0.70), and low degradation rate (31.3 ± 5.9%), in addition to high t_50%_ value of 24 h in SSD in-vitro drug release to accomplish sustained drug release which is comparatively higher than that of commercial SSD cream. The sustained drug release of the thermo-responsive cellulose hydrogel was also confirmed by best fitting of the drug release study results to a zero-order kinetic model. On the other hand, the SSD release mechanism of thermo-responsive cellulose hydrogels followed the non-Fickian diffusion, model, in which SSD release rate is controlled by both SSD drug diffusion and thermo-responsive cellulose hydrogel erosion. The impressive results obtained from this study confirm that thermo-responsive cellulose hydrogels are promising drug delivery media which are able to accomplish sustained drug release reducing the fluctuation of drug levels during administration while using an abundantly available agricultural biomass.

## Figures and Tables

**Table 1:** Formula of thermo-responsive cellulose hydrogel.

Sample	PF127 Polymer (w/v%)	Cellulose Fibers (w/v%)	DI Water (w/v%)
H1	15	0.0	85
H2		1.0	84
H3		2.0	83
H4		3.0	82
H5	20	0.0	80
H6		1.0	79
H7		2.0	78
H8		3.0	77
H9	25	0.0	75
H10		1.0	74
H11		2.0	73
H12		3.0	72
H13	30	0.0	70
H14		1.0	69
H15		2.0	68
H16		3.0	67
H17	35	0.0	65
H18		1.0	64
H19		2.0	63
H20		3.0	62

**Table 2:** SGTT and flow behavior of the thermo-responsive cellulose hydrogels.

Sample	LCST (°C)	UCST (°C)	Status at (4 °C)	Status at (20 °C)	Status at (37 °C)
H1	N/D	N/D	-	-	-
H2	N/D	N/D	-	+	+
H3	N/D	N/D	+	+	++
H4	N/D	N/D	+	++	++
H5	24.0 ± 1.0	58.3 ± 2.9	-	+	+++
H6	23.7 ± 0.6	61.7 ± 2.9	+	+	+++
H7	22.3 ± 1.2	68.3 ± 2.9	+	++	+++
H8	21.0 ± 1.0	78.3 ± 2.9	+	++	+++
H9	20.0 ± 1.0	73.3 ± 2.9	-	+++	+++
H10	17.0 ± 1.0	78.3 ± 2.9	+	+++	+++
H11	15 ± 0.0	86.7 ± 2.9	+	+++	+++
H12	<15	N/D	++	+++	+++
H13	17.7 ± 0.6	N/D	-	+++	+++
H14	15 ± 0.0	N/D	+	+++	+++
H15	<15	N/D	++	+++	+++
H16	<15	N/D	++	+++	+++
H17	16.7 ± 0.6	N/D	-	+++	+++
H18	<15	N/D	+	+++	+++
H19	<15	N/D	++	+++	+++
H20	<15	N/D	++	+++	+++

N/D: not defined; - Liquid free flowing; + High viscosity, slow flow; ++ Weak gel, hard to flow; +++ Solid-like behavior, non-free flow.

**Table 3:** Kinetic and mechanism models’ constants and correlation coefficient, R^2^ of SSD release.

Hydrogel	Zero-Order		First-Order		Higuchi		Korsmeyer-Peppas		
	k_0_	R^2^	k_1_	R^2^	k_H_	R^2^	k_r_	n	R^2^
H5	5.583	0.980	0.140	0.954	26.662	0.968	10.839	0.727	0.994
H6	2.708	0.986	0.048	0.954	15.568	0.955	7.129	0.671	0.991
H7	2.585	0.985	0.041	0.946	14.330	0.923	5.023	0.710	0.967
H8	1.762	0.987	0.023	0.969	10.046	0.940	9.462	0.746	0.976

## Data Availability

Not applicable.

## Supplementary Materials

The following are available online at https://www.mdpi.com/article/10.3390/polym13132153/s1, Figure S1: Schematic illustration of PF127 micelles formation and its interaction with OPEFB extracted cellulose for the synthesis of thermo-responsive cellulose hydrogel. Figure S2: Swelling mechanism of thermo-responsive cellulose hydrogel. Figure S3: Linear regression of kinetic and mechanism models (a) Zero-order model (b) First-order model (c) Higuchi model (d) Korsmeyer- peppas model. Figure S4: SSD release from thermo-responsive cellulose hydrogels by different mechanisms. Table S1: Concentration of SSD released from thermo-responsive cellulose hydrogels at different time interval.
