# Supplementary material for: Green Synthesis of Thermo-Responsive Hydrogel from Oil Palm Empty Fruit Bunches Cellulose for Sustained Drug Delivery
Source: Polymers (Basel). 2021 Jun 29;13(13):2153. doi: 10.3390/polym13132153 (PMC8271751; doi:10.3390/polym13132153)
Supplement: Supplementary file 1 [file polymers-13-02153-s001.zip › polymers-1245247-supplementary.pdf]

Supplementary Material of "GREEN SYNTHESIS OF THERMO-RESPONSIVE HYDROGEL FROM OIL PALM EMPTY FRUIT BUNCHES CELLULOSE FOR SUSTAINED DRUG DELIVERY"

Supplementary figures

Figure S1 Schematic illustration of PF127 micelles formation and its interaction with OPEFB extracted cellulose for the synthesis of thermo-responsive cellulose hydrogel 2
Figure S2 Swelling mechanism of thermo-responsive cellulose hydrogel 3
Figure S3 Linear regression of kinetic and mechanism models (a) Zero-order model (b) First-order model (c) Higuchi model (d) Korsmeyer- peppas model 5
Figure S4 SSD release from thermo-responsive cellulose hydrogels by different mechanisms...... 6

Supplementary tables

Table S1 Concentration of SSD released from thermo-responsive cellulose hydrogels at different time interval 7

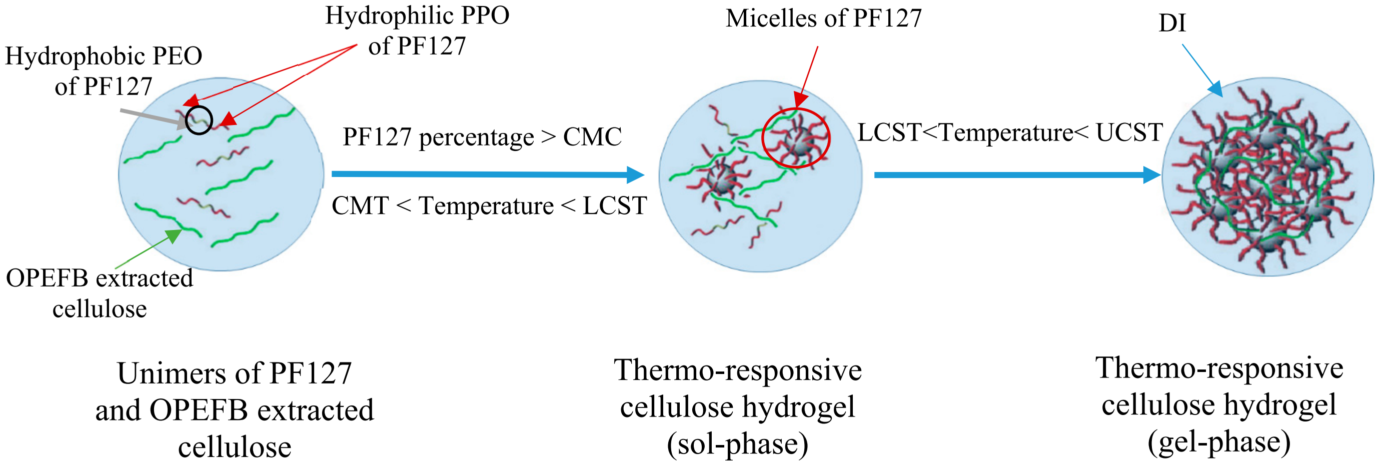

Figure S1 Schematic illustration of PF127 micelles formation and its interaction with OPEFB extracted cellulose for the synthesis of thermo-responsive cellulose hydrogel

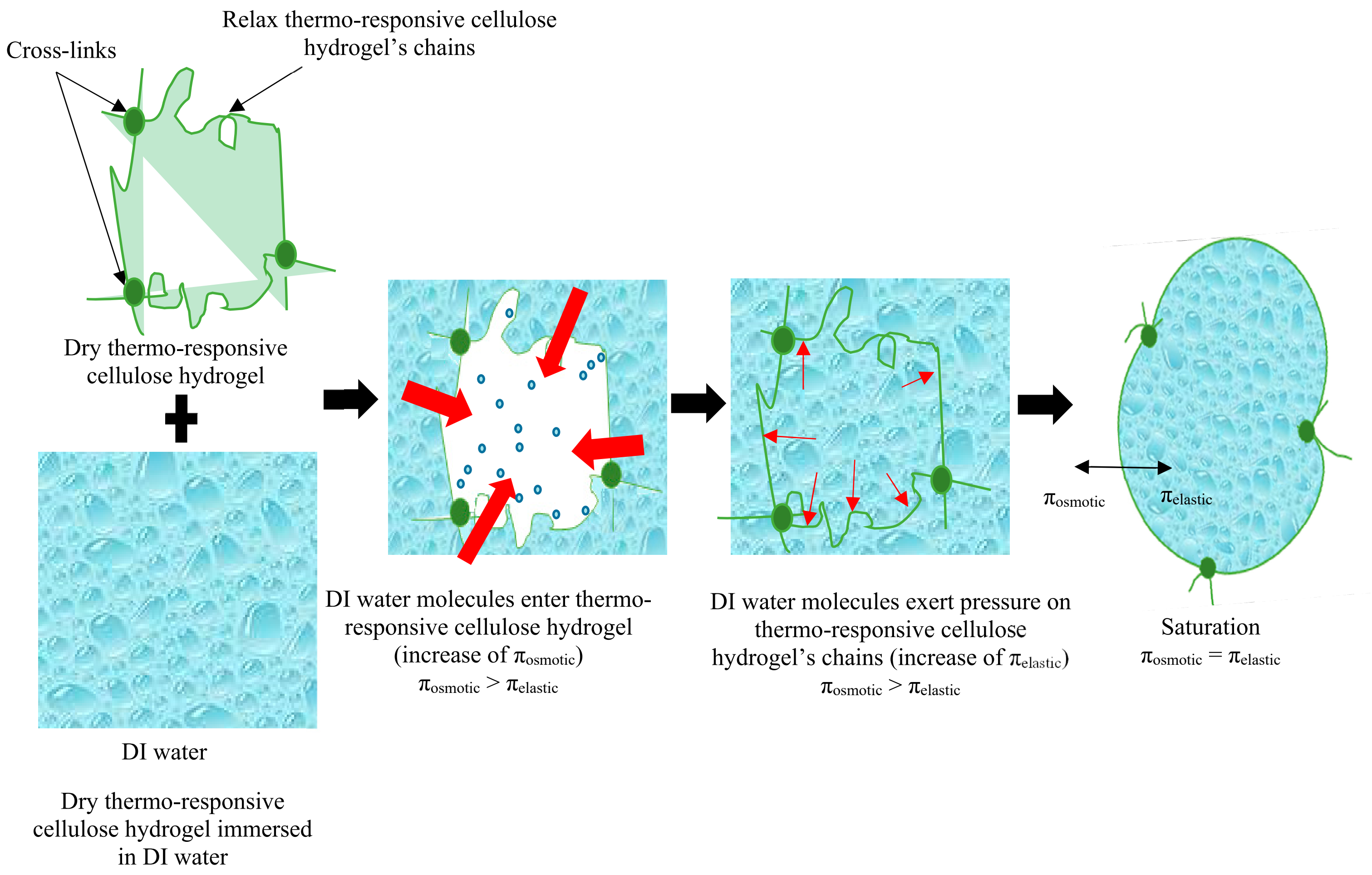

Figure S2 Swelling mechanism of thermo-responsive cellulose hydrogel

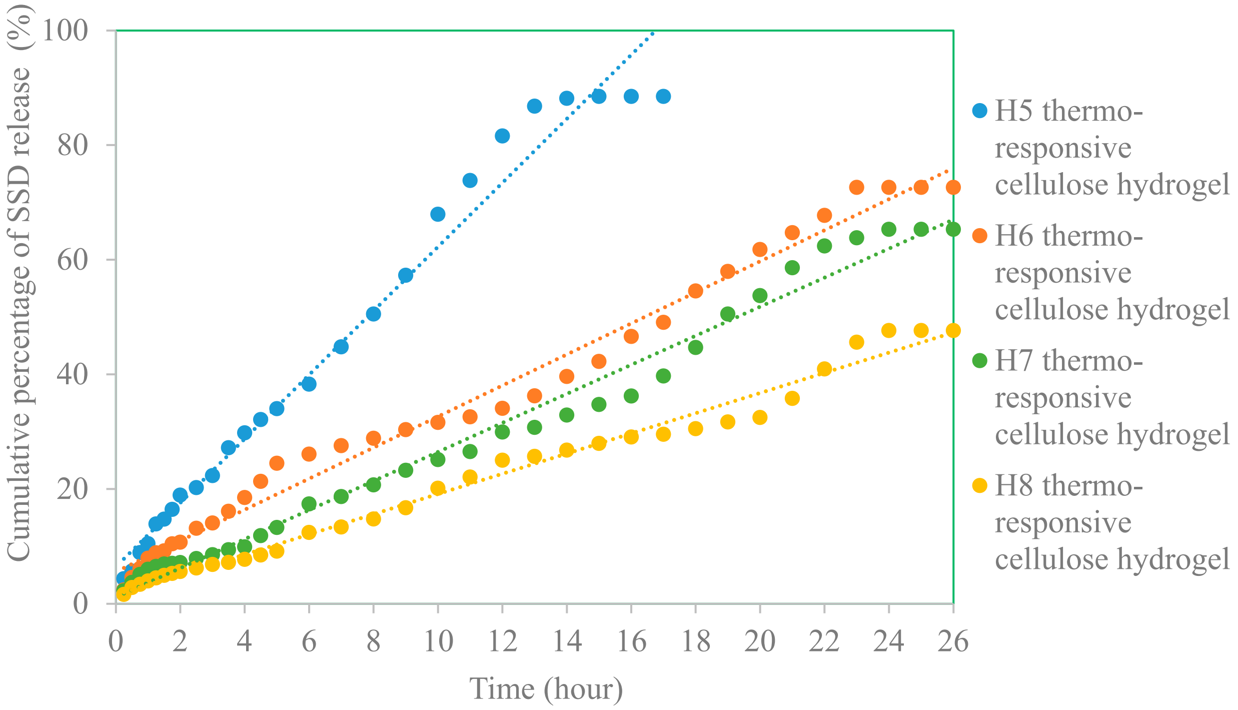

Cumulative percentage of SSD release (%)
100
80
60
40
20
0
0 2 4 6 8 10 12 14 16 18 20 22 24 26
Time (hour)
H5 thermo-responsive cellulose hydrogel
H6 thermo-responsive cellulose hydrogel
H7 thermo-responsive cellulose hydrogel
H8 thermo-responsive cellulose hydrogel

(a)

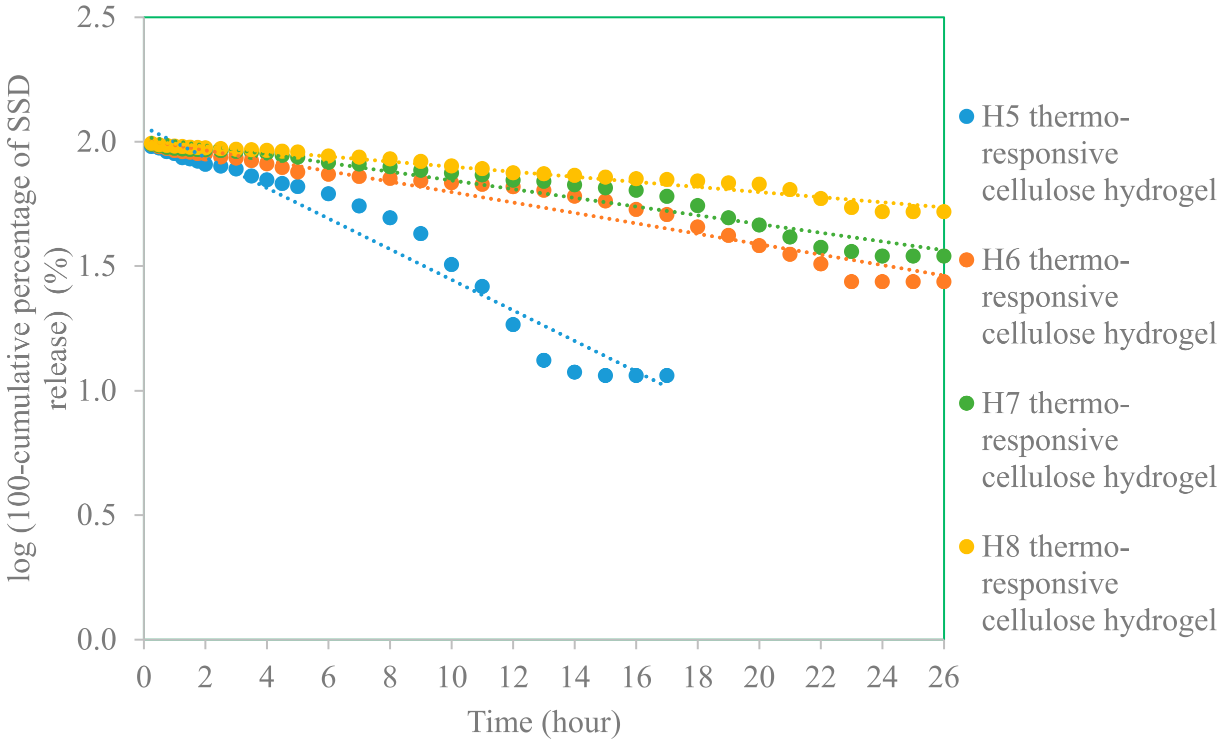

log (100-cumulative percentage of SSD release) (%)
2.5
2.0
1.5
1.0
0.5
0.0
0 2 4 6 8 10 12 14 16 18 20 22 24 26
Time (hour)
H5 thermo-responsive cellulose hydrogel
H6 thermo-responsive cellulose hydrogel
H7 thermo-responsive cellulose hydrogel
H8 thermo-responsive cellulose hydrogel

(b)

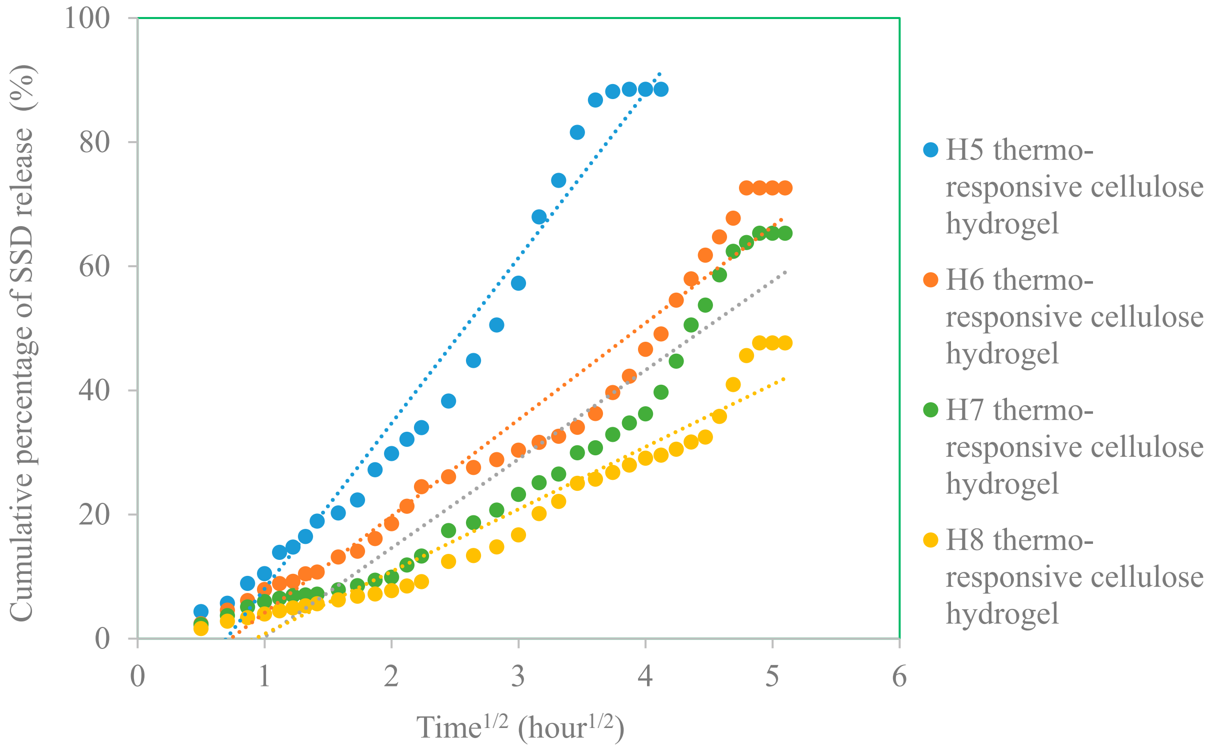

(c)

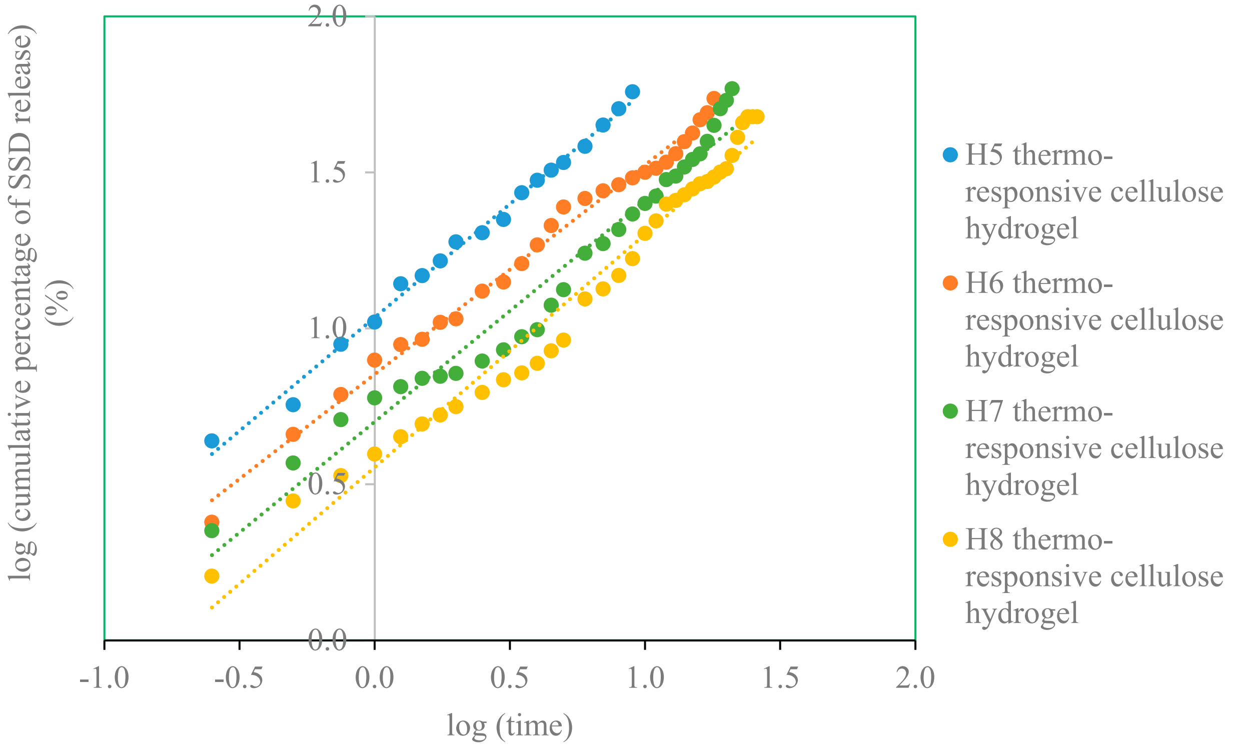

(d)

Figure S3 Linear regression of kinetic and mechanism models (a) Zero-order model (b) First-order model (c) Higuchi model (d) Korsmeyer- peppas model

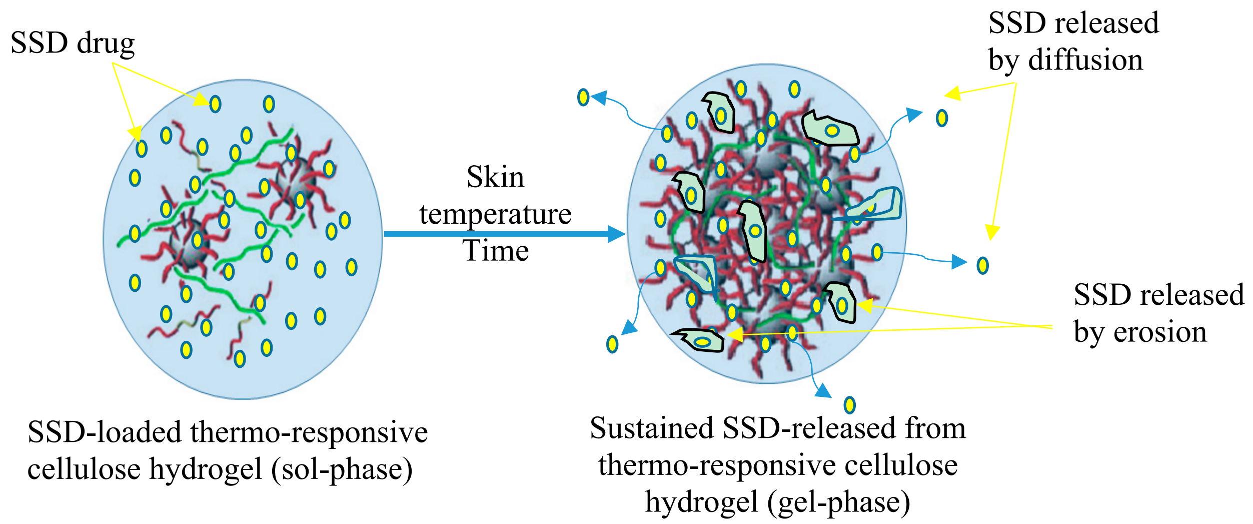

Figure S4 SSD release from thermo-responsive cellulose hydrogels by different mechanisms

Table S1 Concentration of SSD released from thermo-responsive cellulose hydrogels at different time interval

Time (Hour)	Concentration of SSD (mg/L)			
	H5	H6	H7	H8
0.5	9.62	7.91	6.36	4.81
1.0	17.66	13.43	10.09	6.68
2.0	31.90	17.90	11.97	9.38
4.0	50.00	31.17	16.56	12.94
4.5	56.92	41.23	22.34	15.36
6.0	64.42	43.64	29.61	21.16
8.0	84.98	48.24	34.82	24.81
